# Prenatal presentation of an adrenocortical tumor

**DOI:** 10.1002/cnr2.1670

**Published:** 2022-09-02

**Authors:** Jeffrey H. Schwartz, Erlyn Smith

**Affiliations:** ^1^ Department of Pediatrics, College of Medicine University of Florida Gainesville Florida USA; ^2^ UF Health Pediatric Subspecialty Clinic Pensacola Florida USA

**Keywords:** adrenal, adrenocortical, neuroblastoma, suprarenal

## Abstract

**Background:**

Prenatally identified suprarenal masses are most often found to be adrenal hemorrhage. The most common tumor in this situation is neuroblastoma.

**Case Presentation:**

We report the case of a rare adrenocortical tumor found prenatally on ultrasound. While most patients with adrenocortical tumors present with virilizing symptoms, our patient did not have evidence of virilization and was presumed to have neuroblastoma.

**Conclusion:**

Following a period of observation, our patient underwent surgical resection due to tumor growth revealing the unexpected diagnosis.

## 1 INTRODUCTION

Prenatally diagnosed adrenal masses are uncommon.^1^ Most often, these masses are due to an adrenal hemorrhage although tumors and other etiologies occur as well.^2^, ^3^, ^4^, ^5^ Even though imaging may not confirm a definitive diagnosis, surgical intervention is not always required. Adrenocortical tumors are rare pediatric tumors and most patients present in early childhood with signs or symptoms of endocrinopathy.^6^, ^7^ Herein, we report the case of an adrenocortical tumor diagnosed by prenatal ultrasound without associated endocrinopathy.

## 2 CASE PRESENTATION

**FIGURE 1:**
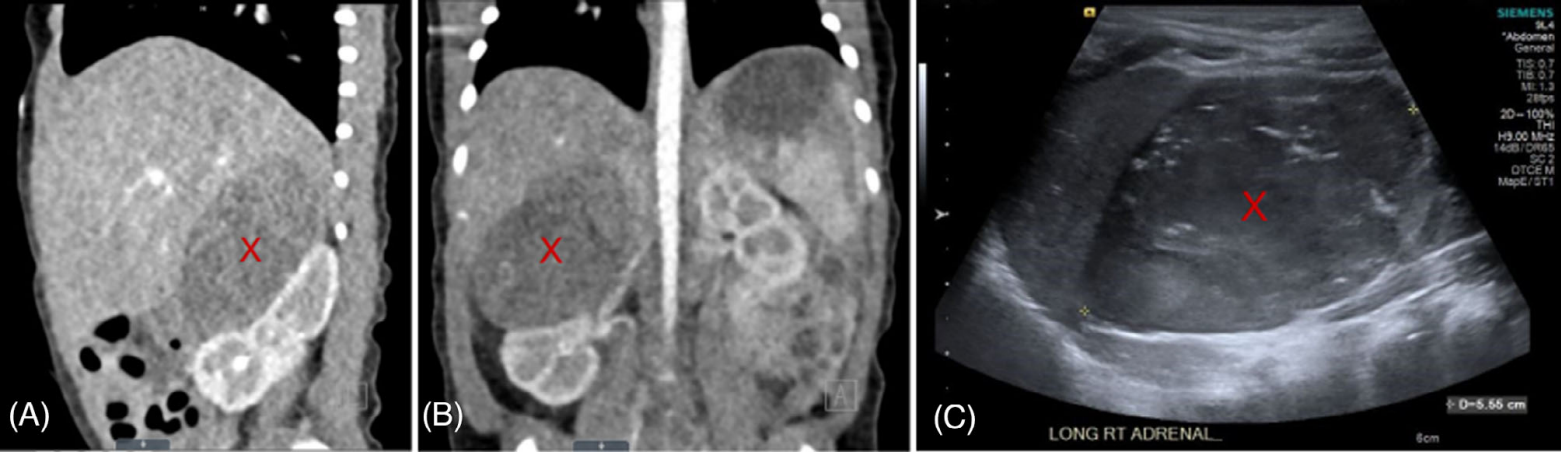
Right adrenal mass (marked with X) shown on initial sagittal CT (A), coronal CT (B), and follow‐up ultrasound (C).

A 3‐day‐old female was transferred from an outside hospital for evaluation of a right adrenal mass. She was born at 39 weeks via repeat Cesarean section. Pregnancy was complicated by maternal preeclampsia and hyperglycemia. Additionally, a right adrenal mass was found on a routine ultrasound 2 weeks prior to delivery. She subsequently had two follow up ultrasounds, one prior to birth and another shortly after birth, which again demonstrated the presence of the right adrenal mass. The abdominal ultrasound showed a 4.8 × 3.9 × 3.3 cm mass that appears confluent to and/or abutting the posterior right kidney. This was followed by a CT abdomen which showed a 4.6 × 3.3 × 3.2 cm right adrenal mass (Figure 1) and the MRI of the abdomen confirmed the presence of the tumor measuring 4.5 × 4 × 3.7 cm.

On exam, the infant was well appearing without any signs of virilization. There was also no evidence of Cushing's syndrome. Her family history was positive only for maternal hypertension; there was no reported history of benign or malignant tumors within the family. Urine catecholamine metabolites were normal: homovanillic acid (HVA) was 13 and vanillylmandelic acid (VMA) was 7. Evaluation for additional adrenocortical hormones was not performed. Metaiodobenzylguanidine (MIBG) scan was negative. She was diagnosed with presumed neuroblastoma without any clinical or radiological evidence of metastatic disease. We followed Children's Oncology Group clinical trial ANBL1232 (off study) of which she was categorized as Group A1 per International Neuroblastoma Risk Group classification (INRG; L1 tumor, age <6 months, tumor size 3.1–5 cm) since she was ineligible for the clinical trial. She was observed every 6 weeks with abdominal ultrasounds and urine catecholamines. At the first 6 week interval, the abdominal ultrasound was unchanged and the urine catecholamine metabolites were slightly increased compared to the initial values though still normal (HVA 19, VMA 10).

At 12 weeks, the adrenal mass increased in size by greater than 50%, measuring at 5.6 × 4.2 × 4.7 cm (Figure 1). Urine catecholamine metabolites were further elevated compared to previous values (HVA 24, VMA 13) although still within normal limits. The parents decided to proceed to tumor resection. The tumor was resected without any complications and pathology showed a 6.3 cm adrenocortical tumor. The tumor was encapsulated with no invasion of the capsule or extension into adjacent structures seen. Focal areas of hemorrhage with necrosis were present. Mitotic figures were ~10 per 20 HPF. Inhibin and Calretinin were both strongly and diffusely positive. Synaptophysin was weakly positive. Chromogranin A was negative and Ki67 showed ~10% to 15% positivity; Chromogranin B was not performed.

## 3 DISCUSSION

The differential diagnosis of a prenatal suprarenal mass includes malignant and benign tumors, as well as nontumor etiologies. The most common etiology is an adrenal hemorrhage. Depending on the case series, neuroblastoma and pulmonary sequestrations are the next most common etiologies.^2^, ^3^, ^4^, ^5^ Prenatal ultrasounds are the standard first mode of imaging. Fetal MRI has been utilized for follow‐up imaging, with one case series showing a 70% concordance rate between ultrasound and MRI.^4^

Postnatal evaluations of adrenal masses frequently include a repeat ultrasound followed by either CT scan or MRI. If imaging does not exclude neuroblastoma, urinary random catecholamine metabolites should be sent, including HVA and VMA. Additionally, an MIBG scan should be obtained. Further management will depend on the underlying etiology.

As our patient had a solid adrenal mass confirmed by CT and MRI, the most likely etiology was felt to be neuroblastoma. Previous studies of neuroblastoma have shown that infants with small localized masses can be observed without intervention.^8^ Of 83 patients observed without intervention, only 19% ultimately underwent surgery. Of those 16 patients, 6 had diagnoses other than neuroblastoma including 2 with adrenocortical tumors. The 3 year Event Free Survival was 97.7% and the 3 year Overall Survival was 100%. A current Children's Oncology Group Clinical trial is investigating expanding the criteria for clinical observation to infants up to 12 months old with INRG Stage L1 adrenal tumors less than 5 cm in greatest diameter. After extensive discussion and review of the literature with the family, the decision was made to observe our patient closely to avoid surgery. Due to the tumor growth and rising catecholamine metabolites (although never elevated per age‐based reference ranges), our patient underwent an uncomplicated resection revealing an adrenocortical tumor.

Adrenocortical tumors have an incidence of 0.2 per 1 000 000 children in the United States according to the Surveillance, Epidemiology, and End Results (SEER) Program.^9^ Most pediatric cases occur in children less than 4 years of age and there is a female predominance.^6^, ^7^ It is uncommon that a diagnosis is made prenatally by ultrasound, with only five previous patients reported to our knowledge.^6^, ^7^, ^10^, ^11^, ^12^ All of the patients with available information had a palpable abdominal mass on exam, unlike our patient who had a normal abdominal exam (Table 1). The majority of patients with adrenocortical tumors has localized disease and present with virilizing symptoms.^6^, ^13^ Our patient had localized disease but did not have virilizing symptoms or other evidence of endocrinopathy. As such, the diagnosis of adrenocortical tumor was not considered likely. Of the adrenocortical tumors, a minority (20%–33%) is adenomas^9^, ^14^ and differentiation between adenoma and carcinoma in pediatric patients can be challenging.^7^ A study by Wieneke et al. proposed a scoring system which accurately predicts benign versus aggressive tumor behavior.^15^, ^16^, ^17^ Our patient's tumor met only one of the Wieneke criteria definitively (atypical mitosis) with an additional criterion being suggestive (focal vs. confluent necrosis), favoring a benign clinical course. An additional report by Picard et al. showed an association between Ki67 index positivity (>15%) and worse clinical outcomes.^18^ Our patient's Ki67 index positivity was ~10%–15%. A comparison between our patient's tumor's pathologic features and published features associated with worse clinical outcomes is shown in Table 2. Additionally, younger age and localized disease are associated with an improved prognosis.^6^ Complete resection is the standard of care whereas adjuvant chemotherapy has not shown added benefit, particularly in advanced disease.^6^, ^19^

**TABLE 1:** Clinical features at birth of patients with adrenocortical tumors diagnosed prenatally

Series	Gender	Postnatal clinical findings
Godil et al.	M	Abdominal mass, seizure, pulmonary metastases
Izbizky et al.	F	Abdominal mass, cliteromegaly
Michalkiewicz et al.	NR	NR
Miele et al.	NR	NR
Sarwar et al.	M	Abdominal mass
Present case	F	None

Abbreviations: NR, not reported.

**TABLE 2:** Pathologic features of adrenocortical tumors associated with worse clinical outcomes

Pathologic feature	Published criteria	Our patient's results
Tumor weight	>400 g	136 g^a^
Tumor size	>10.5 cm	6.3 cm
Vena cava invasion	Present	Absent
Capsular and/or vascular invasion	Present	Absent
Extension into perirenal soft tissue	Present	Absent
Confluent necrosis	Present	Focal areas of necrosis
Severe nuclear atypia	Present	Absent
>15 Mitotic figures per 20 HPF	Present	10 Mitotic figures per 20 HPF
Atypical mitotic figures	Present	Present
Ki67 index positivity	>15%	~10%–15%

*Note*: All criteria published by Wieneke et al. except (Ki67 index positivity) published by Picard et al.

^a^ Weight not documented; estimated based on pre‐surgical imaging measurements.

Adrenocortical tumors, while rare, are frequently associated with cancer predisposition syndromes. Germline mutations have been identified in the TP53 gene on chromosome 17 in up to 82% of patients with adrenocortical tumors.^20^, ^21^ There are almost 250 mutations associated with this gene that cause Li‐Fraumeni syndrome, an autosomal dominant condition leading to increased risk of many types of cancers.^22^ Germline mutations have also been discovered in chromosome 11p15 leading to paternal uniparental disomy, as can be seen in Beckwith‐Wiedemann syndrome.^23^ This report by Pinto et al. found that 6/6 patients with adrenocortical tumors and no TP53 mutation had 11p15 mutations. It is important to test all patients with adrenocortical tumors for cancer predisposition syndromes as there is not always a family history and ongoing tumor surveillance may be indicated. Our patient had no family history of cancer and tested negative for TP53 and 11p15 mutations. She is currently being monitored with history, exam, and abdominal ultrasound every 3 months.

In summary, our patient presented with a solid adrenal mass in utero. Her clinical presentation was atypical for prenatally diagnosed adrenocortical tumor and more consistent with prenatal neuroblastoma, despite having a negative MIBG and normal urine catecholamines. She underwent a complete resection at 3½ months of age due to tumor growth revealing an adrenocortical tumor with predicted benign activity. This case exemplifies the importance of maintaining a broad differential diagnosis and close observation when there is not a definitive diagnosis.

## AUTHOR CONTRIBUTIONS

**Jeffrey H. Schwartz:** Conceptualization (lead); investigation (supporting); methodology (equal); writing – original draft (lead); writing – review and editing (equal). **Erlyn Smith:** Conceptualization (supporting); investigation (lead); methodology (equal); writing – original draft (supporting); writing – review and editing (equal).

## CONFLICT OF INTEREST

The authors have stated explicitly that there are no conflicts of interest in connection with this article.

## ETHICS STATEMENT

Institutional approval was not required for this single patient case report. Informed consent for publication was obtained from the patient's parents for the publication of case details and use of images.

## Data Availability

Data sharing is not applicable to this article as no new data were created or analyzed in this study.
